# P-1942. Candidozyma auris Susceptibility from Michigan Hospitals to Rezafungin and Nine Other Antifungal Agents

**DOI:** 10.1093/ofid/ofaf695.2110

**Published:** 2026-01-11

**Authors:** Katherine Klamer, Ashish Bhargava, Leonard Johnson, Mamta Sharma, Louis Saravoltaz

**Affiliations:** Henry Ford Health System, Grosse Pointe Woods, MI; Henry Ford St. John Hospital, Grosse Pointe Woods, Michigan; Henry Ford St. John Hospital, Grosse Pointe Woods, Michigan; Henry Ford Health System, Grosse Pointe Woods, MI; Henry Ford Health System, Grosse Pointe Woods, MI

## Abstract

**Background:**

*C. auris* has been reported as a multidrug resistant pathogen with variability of susceptibility to antifungal agents. Multiple clades have been identified with different geographic penetrance. In view of the severity of illness and mortality associated with *C. auris* infections, optimal therapy needs additional guidance in terms of in vitro data and identification of new antifungal agents. Our study evaluated in vitro activity of ten antifungal agents against 67 isolates from Michigan and compared them to isolates from sites outside of Michigan.

**Figure:**
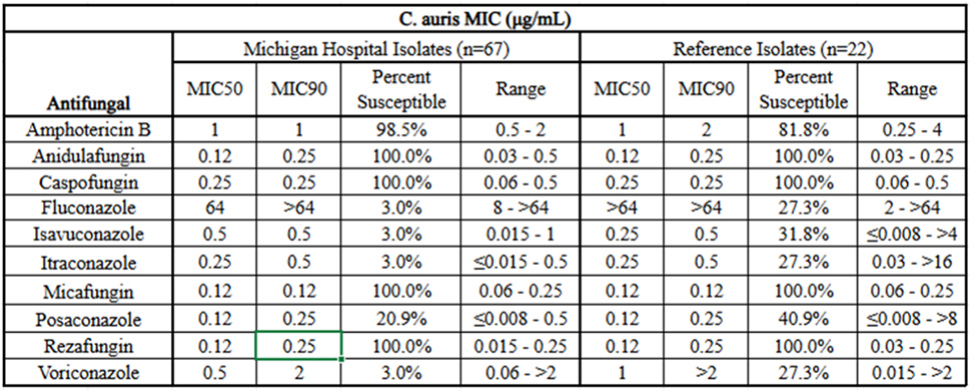
C. auris MIC

**Methods:**

*C. auris* isolates were processed on sabouraud dextrose agar at 35°C for 24 hours and identification was performed by MALDI-TOF-MS or by Vietk-2 yeast card. Identity was confirmed by CHROMagar Candida Plus. The antifungals tested were amphotericin B, anidulafungin, caspofungin, fluconazole, isavuconazole, itraconazole, micafungin, posaconazole, rezafungin and voriconazole.

Broth microdilution antifungal susceptibility testing (AST) was performed according to Clinical Laboratory Standards and Antifungal Susceptibility Testing of Yeasts. AST testing was performed using Sensititre™ YeastOne™ AST Plate.

Percent sensitivity was determined by tentative breakpoints from the CDC *C. auris* breakpoint guidelines, CLSI M23M44 Ed3, EUCAST antifungal breakpoints v 11.0 and estimated breakpoints from published reports.

**Results:**

*In vitro* testing results are summarized in table 1. The amphotericin B susceptibility was higher for Michigan isolates than those from sites outside of Michigan (98.5% vs 81.8%) and each of the azoles demonstrated less activity against the Michigan hospital isolates vs the reference isolates. All the echinocandins demonstrated excellent activity against strains whether they were present in Michigan or outside of Michigan.

**Conclusion:**

*C. auris* demonstrated differences in susceptibility from Michigan hospitals by being more susceptible to Amphotericin B and less susceptible to the azoles than the isolates from other areas of the country. All isolates were susceptible to the echinocandins tested. Regional susceptibility testing of *C. auris* should be considered because of variability among clades and therapeutic options that clinicians need to have when treating serious *C. auris* infections.

**Disclosures:**

All Authors: No reported disclosures

